# Effect of New Zealand blueberry consumption on recovery from eccentric exercise-induced muscle damage

**DOI:** 10.1186/1550-2783-9-19

**Published:** 2012-05-07

**Authors:** Yanita McLeay, Matthew J Barnes, Toby Mundel, Suzanne M Hurst, Roger D Hurst, Stephen R Stannard

**Affiliations:** 1 School of Sport and Exercise, Massey University, Palmerston North, New Zealand; 2 Food and Wellness Group, The New Zealand Institute for Plant and Food Research Ltd., Private Bag 11030, Palmerston North, New Zealand

## Abstract

**Background:**

Exercise-induced muscle damage (EIMD) is accompanied by localized oxidative stress / inflammation which, in the short-term at least, is associated with impaired muscular performance. Dietary antioxidants have been shown to reduce excessive oxidative stress; however, their effectiveness in facilitating recovery following EIMD is not clear. Blueberries demonstrate antioxidant and anti-inflammatory properties. In this study we examine the effect of New Zealand blueberries on EIMD after strenuous eccentric exercise.

**Methods:**

In a randomized cross-over design, 10 females consumed a blueberry smoothie or placebo of a similar antioxidant capacity 5 and 10 hours prior to and then immediately, 12 and 36 hours after EIMD induced by 300 strenuous eccentric contractions of the quadriceps. Absolute peak and average peak torque across the knee, during concentric, isometric, and eccentric actions were measured. Blood biomarkers of oxidative stress, antioxidant capacity, and inflammation were assessed at 12, 36 and 60 hours post exercise. Data were analyzed using a two-way ANOVA.

**Results:**

A significant (*p* < 0.001) decrease in isometric, concentric and eccentric torque was observed 12 hours following exercise in both treatment groups. During the 60 hour recovery period, a significant (*p* = 0.047) interaction effect was seen for peak isometric tension suggesting a faster rate of recovery in the blueberry intervention group. A similar trend was observed for concentric and eccentric strength. An increase in oxidative stress and inflammatory biomarkers was also observed in both treatment groups following EIMD. Although a faster rate of decrease in oxidative stress was observed in the blueberry group, it was not significant (*p* < 0.05) until 36 hours post-exercise and interestingly coincided with a gradual increase in plasma antioxidant capacity, whereas biomarkers for inflammation were still elevated after 60 hours recovery.

**Conclusions:**

This study demonstrates that the ingestion of a blueberry smoothie prior to and after EIMD accelerates recovery of muscle peak isometric strength. This effect, although independent of the beverage’s inherent antioxidant capacity, appears to involve an up-regulation of adaptive processes, i.e. endogenous antioxidant processes, activated by the combined actions of the eccentric exercise and blueberry consumption. These findings may benefit the sporting community who should consider dietary interventions that specifically target health and performance adaptation.

## Background

Strenuous eccentric muscular work is common in many sporting events, particularly those which involve jumping, changing direction/stopping at speed, rapid acceleration and being pushed upon by opposing players. Training and competition in field and court-based team sports therefore will necessitate eccentric muscle contraction which, depending on intensity and duration, may bring about various levels of damage to contractile and connective tissue components of skeletal muscle [1,2]. This damage is typically associated with impaired muscle function, inflammation, pain, localised swelling/edema, and leakage of myofibril proteins [3,4]. These effects, particularly impaired muscle function and pain, may negatively impact performance during successive games (common during tournament competition), or the athletes’ ability to train during the following days [5,6]. Importantly, if the ability to train is impaired, adaptation and therefore subsequent performance improvements may be delayed.

Although the mechanisms behind exercise-induced muscle damage (EIMD) are not precisely known it is believed that along with initial mechanically-induced disruption of the extracellular matrix, sarcolemma, sarcoplasmic reticulum, t-tubules and contractile proteins, secondary damage is caused by the production of reactive oxygen species (ROS) at the site of injury by phagocytic cells [7]. Degradation of muscle tissue, through a combination of phagocytosis, protease production and the release of cytotoxic and cytolytic molecules, such as superoxide [8], is believed to contribute further to the already lowered force generating ability of the effected muscle fibres [9,10].

The efficacy of dietary antioxidant supplementation in facilitating recovery following strenuous muscle damaging exercise is under debate. While it is well understood that antioxidants play a pivotal role in countering free radical activity within the body, research investigating classical antioxidant supplementation (such as vitamin C and E) on the rate of recovery from EIMD, particularly functional recovery, has consistently shown little or no benefit from supplementation [11-14]. Blueberry fruit are normally consumed as a whole fruit (fresh or frozen) and although they are low in vitamin C and E they contain the broadest range of anthocyanin and polyphenolic antioxidant compounds among common berryfruits [14]. Blueberry fruit exhibit a high antioxidant capacity (oxygen radical absorption capacity - ORAC) and have been shown to reduce oxidative stress and inflammation [15,16] indicating that blueberry-derived anthocyanins may modulate cellular events independent of the fruit’s inherent antioxidant capacity [17-20]. These findings suggest that supplementation with these polyphenolic-rich fruit may help reduce secondary damage and therefore minimize EIMD related changes in muscle performance and soreness. The aim of this study therefore was to investigate the effect of blueberry consumption on markers of EIMD and inflammation after strenuous eccentric exercise.

## Methods

### Subjects

Ten healthy females (22 ± 1 years; 62 ± 8 kg; 167 ± 5 cm) were recruited via word-of-mouth to participate in this study. All subjects were physically active and participated in recreational level resistance and aerobic based exercise at least twice per week. All subjects had at least one years’ experience in training in this manner. Subjects filled out a Health Screening Questionnaire to exclude those who were at risk physically, culturally, or religiously in following the protocol. Those who passed the questionnaire were asked to give written consent. Approval for this study was granted by the local Human Ethics Committee (09/73).

### Study design

This was a balanced, randomized crossover design where the response to the treatment trial (blueberry condition) was measured as the performance of one leg and, on another occasion, the response to the control condition was measured as the performance of the contralateral leg. The two experimental trials were separated by at least a month, dependent on the individual’s menstrual cycle.

### Experimental protocol

*Familiarization session.* During the week preceding the first trial, subjects attended a familiarization session in which they carried out the required movements that were to be used for performance testing on a Biodex isokinetic dynamometer (Biodex Medical Systems Inc., NY). Appropriate seat positions were determined using recommendations made by the manufacturer (Biodex Medical Systems Inc., 2004) and were recorded for subsequent use throughout the study. Menstrual cycle was also recorded in order to test the subjects during the luteal phase (day 14 until day 1 of next period) of each trial. This was done so that hormone levels and body temperature were similar in both trials. Subjects were asked to abstain from any form of exercise apart from necessary walking 48 hours prior to and until 60 hours post trial.

*Day of trial.* On the day of the trial, subjects were required to attend the laboratory in the morning where blood was withdrawn by venipuncture into appropriate tubes for plasma and serum separation, which was then frozen (−20°C) in aliquots for biochemical analysis. They were then asked to complete a 5 minute warm up on a Monark cycle ergometer before pre-damage performance testing was carried out. This involved five maximal efforts each of isometric, concentric and eccentric contractions of the quadriceps muscle while seated on an isokinetic dynamometer. Once completed, the subjects were given their allocated beverage (blueberry treatment or control) and were required to return to the laboratory around midday for a standardized lunch, which included muesli bars and a repeat of their allocated beverage. They returned a third time in the evening for repeated blood sampling, warm-up, and the eccentric bout of exercise which involved 300 maximal eccentric repetitions using the quadriceps muscles to elicit muscle damage. Dietary intervention and selection of leg exposed to eccentric exercise were randomly allocated between subjects. Subjects returned to the laboratory the following three mornings (12, 36, and 60 hours post-damage) for follow up blood samples, performance tests, ratings of muscle soreness, and a standardized breakfast which included their allocated beverage.

### Dietary intervention

On the day of eccentric muscle damaging exercise subjects were required to attend the laboratory in the morning, around midday and in the evening. On each occasion, an allocated beverage (blueberry treatment or control) was consumed along with a “liquid breakfast” drink (Sanitarium ‘Up & Go^TM^, New Zealand Health Association Ltd, Auckland, New Zealand) in the morning, and muesli bars (Tasti Products Ltd, Auckland, New Zealand) at midday, 10 hours and 5 hours, respectively, before the onset of the eccentric muscle damaging exercise. In the evening, control or blueberry beverage was consumed immediately post-damage along with a standardized meal of rice and curry. Subjects were asked to avoid consuming any other food during that day additional to what was provided. This allowed for a full 24 hours of standardized food intake. Control or blueberry beverage were then given at 12 hours and 36 hours post-muscle damage and coincided with performance and blood measurements. No treatment was given 60 hour post-damage. Each treatment smoothie blended 200 g frozen New Zealand blueberries (cultivar “Maru”), a banana (~ 50 g) and 200 mL commercial apple juice (“Fresh Up^TM^”, Frucor beverages Ltd., Auckland, New Zealand). The control beverage omitted blueberries for 25 g dextrose, required to make control and treatment isocaloric. Table 1 displays the composition of the beverages where although vitamin C, E and the antioxidant capacity (determined by ORAC) for the placebo and blueberry beverages are similar, the blueberry beverage contains over five times more polyphenolic compounds than the placebo of which anthocyanins are the primary component. Over the course of the trial, subjects consumed a total of 1 kg of New Zealand blueberries. For the duration of the first trial, from immediately post-exercise until 60 hours post-muscle damage, subjects were asked to keep a food record so that a similar diet could be followed during the second trial. They were also provided with a list of foods and beverages, including those high in antioxidants, to avoid during each trial. Subjects were regularly reminded of the importance of replicating their diet between trials and of avoiding the specified foods and beverages.

**Table 1:** Antioxidant capacity and content of beverages

	Placebo	Blueberry
ORAC (*μmol trolox equ*)	5298	5417
Total phenolics (*mg/Gallic acid equ.*)	29	168
­Anthocyanins (*mg*)	0	96.6
­Phenolic acid (*mg*)	0.6	26
­Flavanoids (*mg*)	0	10.2
Vitamin C (*mg*)	39.5	45
Vitamin E (*mg*)	1	3

Values reported per 100 mL beverage.

The amount of blueberry fruit used in each serving (200 g) was based upon a similar study by Serfini *et al.*[21], but also considered palatability, avoidance of gastrointestinal upset (often occurring with high intake of fructose found in fruit), and the possibility of hypoglycemia later during the day. Timing and frequency of intake (3 times on day of damage; one with each meal, and 1 each morning for the following two mornings) was decided more for the sake of convenience with subjects coming to the laboratory in the morning for performance and blood measures taken whilst post-absorptive.

### Eccentric (muscle damaging) exercise

The range of motion was set at 60° from maximal knee flexion (0°) to 60° extension (using the dynamometers inbuilt goniometer), with repetitions being performed at an angular velocity of 30°/sec a range and speed proven to effectively bring about a high level of muscle damage and subsequent soreness [22]. Subjects performed 3 sets of 100 eccentric repetitions of the quadriceps muscle. Each set was separated by 5 minutes of passive recovery during which time subjects remained seated on the dynamometer and were allowed water. During the sets subjects were encouraged to exert maximal effort through the full range of motion, resisting the downward pull of the dynamometer arm. The torque they produced was displayed on the computer screen to which they had full visual access during the duration of the exercise.

### Muscle function testing

Subjects were required to complete a 5 minute warm-up on a bicycle ergometer (Monark, Varberg, Sweden) at 100 W prior to all performance tests. Upon completion, the subject was seated on the isokinetic dynamometer at the previously recorded seat adjustments so that the femoral epicondyle was aligned with the dynamometer's axis of rotation and the ankle strap positioned 5 cm proximal to the medial malleolus. Along with the ankle, straps were placed around the chest, hips and the leg to be tested in order to isolate the quadriceps muscle. Range of motion of the leg was set at 60° for concentric and eccentric contractions, and at 75° for isometric contractions, which allowed the weight of the leg to be determined. The subject then performed 5 maximal contractions of each type with each set separated by two minutes of passive recovery. Concentric and eccentric torque was measured at an angular velocity of 30 /sec [8]. Absolute peak torque/tension (PT); the peak torque out of the 5 contractions and average peak torque/tension (APT); the average peak torques taken from the 5 contractions were recorded.

### Perceived muscle soreness

On the three mornings following damage (12, 36 and 60 hours post), subjects’ ratings of perceived muscle soreness were recorded using a subjective scale from 0 to 10 (0 = no soreness, 10 = very, very painful) [23] . Subjects were asked to step up (concentric muscle action) onto a 40 cm box then step down (eccentric muscular contraction) and the soreness in doing so was rated. The three scales (for the three mornings) were all contained on one sheet of paper, but marked soreness values from preceding mornings were covered on the second and third mornings to avoid comparison by the subject.

### Biochemical analyses

*Creatine kinase.* Analysis of the muscle damage marker creatine kinase (CK), in serum collected before and 12, 36 and 60 hours post damage, was carried out at a commercial blood testing laboratory (MedLab Central, Palmerston North, New Zealand). An enzymatic ‘reverse reaction’ method was employed, which photometrically measures the rate of NADPH formation as a final product of the last of three reactions, to quantify CK activity. Results are expressed as % change from pre-damage levels.

*Plasma protein carbonyls.* Plasma protein carbonyls were measured using the method previous described by Levine *et al.*[24]. Briefly, 50 μL of plasma was added to an equal volume of 2,4-dinitro-phenylhydrazine (DNPH, Sigma-Aldrich, Auckland, New Zealand) in 2 M HCl (control = DNPH/HCl in the absence of plasma) and incubated in the dark for 1 hour. Protein was precipitated with 50% trichloroacetate (TCA, Sigma-Aldrich, Auckland, New Zealand) and the pellet washed three times with ethanol:ethylacetate (1:1). The pellet was then re-suspended in 1 mL 6 M guanidine hydrochloride (Merck NZ Ltd., Palmerston North, New Zealand) at 37°C for approximately 15 min, followed by the absorbance being measured at 360 nm in a UV-visible 1601 spectrophotometer (Shimadza Corporation, Kyoto, Japan). Protein carbonyl levels were then calculated from the absorbance difference between test and control using the molar absorption coefficient (ϵ): 22,000 M^-1^ cm^-1^. Plasma protein levels were measured using the Bradford method [25] using commercial Bradford reagent (BioRad Laboratories). Results are calculated as nmol of protein carbonyls/mg total protein and expressed as % change from pre-damage levels.

*Plasma radical oxygen species (ROS)-generating potential*. Hydrolysed carboxy-dihydro-2′,7′-dichlorohydrofluorescein diacetate (carboxy-H_2_DCFDA, Merck, Ltd., Palmerston North, New Zealand) was used to assess the ROS-generating capacity of plasma, using a method previously described by Hurst *et al.*[26]. Briefly, dihydro-2′,7′-dichlorohydrofluorescein (DCF), which is fluorescent when oxidised was added to diluted (1:4) plasma collected pre and post damage at 12, 36 and 60 hours in phosphate buffered saline [PBS], pH 7.4, Invitrogen NZ Ltd., Auckland, New Zealand), or PBS control, then 0.25 μM H_2_O_2_ was added and the changes in fluorescence measured over 1600 s at 22°C using a fluorescence plate reader (BMG FluorStar Optima, Alphatech Systems Ltd, Auckland, New Zealand) with excitation and emission wavelengths of 485 and 520 nm respectively. Controls consisted of PBS/0.25 μM H_2_O_2_ in the absence (control) or presence of diluted plasma. Data were calculated as a change in fluorescence over time (900 s) minus the fluorescence observed at time zero (ΔFI). Results are calculated as ΔFI after 300 sec and shown as % change from pre-damage values.

*Plasma interleukin (IL)-6.* Plasma (100 μL), collected pre and 12, 36 and 60 hours post damage was measured for IL-6 using a sandwich ELISA, purchased from R&D Systems, (Minneapolis, MN, USA).

*Plasma antioxidant capacity.* The capacity to reduce ferric ions was determined using the ferric reducing antioxidant power (FRAP) assay as described by Benzie and Strain [27]. Briefly, an aliquot of 8.5 μL of normal (non- deproteinized) serum was added to 275 μL of diluted FRAP reagent (pre-warmed to 37°C) using a microplate and the plates were incubated at 37°C for 30 mins before measuring the absorbance at 595 nm using a plate reader (ELX 808 Ultra Microplate Reader (Bio-tek Instruments. Inc, USA)). The working FRAP reagent was prepared by mixing 10 volumes of 300 mmol/L acetate buffer, pH 3.6, with 1 volume of 10 mmol/L TPTZ (2,4,6-tripyridyl-s-triazine) in 40 mmol/L hydrochloric acid and with 1 volume of 20 mmol/L ferric chloride. A standard curve was prepared using different concentrations (200–2000 μmol/L) of FeSO_4_.7H_2_O. FRAP was calculated and expressed as either μmol/L or % of pre-treatment values.

### Statistical analyses

Data were analyzed using Statistical Analysis Software (SAS) 9.1 for Windows (version 5.1.2600). Using a repeated measures analysis of variance (ANOVA), comparison between conditions (blueberries and control) over time for each measure (independent variable) were determined, providing levels of significance for Trial effect, Treatment effect, and interaction effect between Treatment and Trial. Where significance permitted, post-hoc tests were performed to identify significant differences at each time point. Represented values are means ± standard deviation (or standard error) for *n* = 10 at a 95% significance level (*p* = 0.05). Paired t-tests were used to determine order effects for performance measures and effort during the 300 maximal eccentric contractions of the quadriceps. Pearson’s Product Moment Correlation Coefficient’s were determined using SPSS 15.0 for Windows. This allowed us to investigate any relationships between certain variables (i.e. antioxidant activity with muscle performance measures) by giving an r-value between 0.0 and 1.00 (or −0.0 and −1.00).

## Results

### Intervention diet

All subjects completed the study and there were no reported adverse effects from the dietary intervention.

### Performance (muscle function)

**Figure 1:**
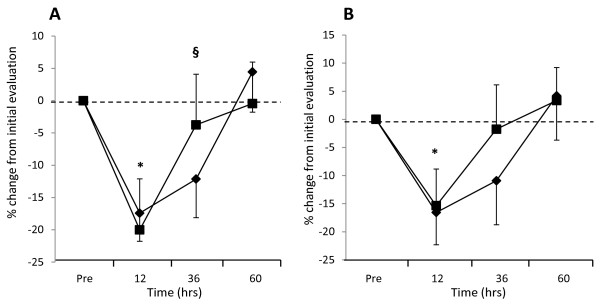
**Isometric torque evaluation after strenuous exercise. [A]** Peak and **[B]** Average isometric torque were assessed pre and 12, 36 and 60 hours after 300 eccentric contractions of the quadriceps under control (♦) or blueberry (■) conditions. Results are expressed as mean ± standard error of percentage change from initial performance evaluation, n = 10 volunteers. * *P* < 0.001 represents significant difference from initial performance evaluation and § *P* < 0.05 represents significant treatment (blueberry) x time interaction, n = 10 volunteers.

Overall changes in volunteer’s physical performance following a strenuous exercise designed to cause muscle damage were evaluated by measuring the torque generated during a series of isometric, eccentric and concentric exercises over a 60 hour recovery period (Table 2). Performance data were normalized relative to pre-damage measures and assessed as a percent (%) change in performance recovery as well as account for leg strength differences (even the control and blueberry treatment conditions were balanced). Significant differences in % change from pre-damage evaluation in peak and average isometric tension, concentric torque, and eccentric torque were seen between time points (*p* < 0.001). The percentage decrease in isometric, concentric and eccentric torque/tension from pre-damage values did not significantly differ between conditions: blueberry treatment decreased in peak torque by 20, 24, and 21% (isometric tension, concentric and eccentric torque) respectively, and the control by 17, 28, and 20% respectively. Similar percentage decreases (and non-significant differences) were seen in average peak torque/tension for blueberry (16, 24 and 16%) and control (17, 24 and 20%) for isometric, concentric and eccentric measures respectively. This type of decrease would be expected, given that 300 strenuous eccentric contractions should bring about maximal fatigue and damage to the quadriceps muscles. Return to pre-damage performance capability was observed by 60 hours recovery in both blueberry and control conditions. A significant interaction effect was seen between time and treatment for peak isometric tension (*p* = 0.047) indicating a faster rate of recovery with the blueberry beverage in the first 36 hours (Figure 1A). Improvements in performance, after 36 hours recovery, were also observed in peak concentric and eccentric torque with blueberries compared with the control (placebo) condition, however, no significant interaction effect was observed between time and treatment (*p* = 0.564 and 0.578 respectively). Similar trends were also observed in evaluating average isometric (Figure 1B), concentric and eccentric torque, again with no significant interaction between time and treatment being observed (*p* = 0.597, 0.449 and 0.880 respectively).

**Table 2:** Changes in muscular performance and perceived soreness following eccentric exercise

	**Peak torque (Nm)**			**Average torque (Nm)**		
	**PLA**	**BB**	**statistical analysis**	**PLA**	**BB**	**statistical analysis**
**ISO**						
Pre	159.25 ± 35.12	173.78 ± 38.52	Time effect, P < 0.001*	142.80 ± 38.19	153.69 ± 36.02	*Time effect, P = 0.511*
12 h	*131.14* ± 33.56	133.91 ± 30.78	*Treatment effect, P = 0.943*	118.14 ± 37.02	128.02 ± 30.25	*Treatment effect, p = 0.597*
36 h	*140.25* ± 43.58	161.73 ± 29.63	Interaction, P = 0.047§	126.15 ± 45.01	146.18 ± 30.16	*Interaction, P = 0.597*
60 h	164.93 ± 40.52	168.52 ± 26.77		144.83 ± 37.58	156.77 ± 29.15	
**CON**						
Pre	145.64 ± 30.89	155.55 ± 23.37	*Time effect, P < 0.001*	131.56 ± 29.23	143.88 ± 22.80	Time effect, P < 0.001*
12 h	106.97 ± 27.49	117.64 ± 20.29	*Treatment effect, P = 0.376*	96.16 ± 29.81	108.91 ± 21.23	*Treatment effect , P = 0.449*
36 h	112.67 ± 35.36	124.91 ± 28.81	*Interaction, P = 0.564*	99.91 ± 33.20	114.85 ± 26.26	*Interaction, P = 0.578*
60 h	130.75 ± 38.07	136.21 ± 31.86		116.25 ± 34.08	126.25 ± 28.08	
**ECC**						
Pre	192.18 ± 46.51	210.38 ± 44.06	Time effect, P < 0.001*	173.81 ± 43.04	188.50 ± 52.26	Time effect, P < 0.001*
12 h	150.31 ± 28.15	162.71 ± 26.89	*Treatment effect, P = 0.840*	135.90 ± 26.04	149.49 ± 23.45	*Treatment effect, P = 0.221*
36 h	157.01 ± 44.63	179.57 ± 31.84	*Interaction, P = 0.426*	145.94 ± 40.77	162.04 ± 31.27	*Interaction, P = 0.88*
60 h	179.03 ± 44.99	189.82 ± 34.55		164.21 ± 44.46	176.86 ± 33.19	
	**Perceived muscle soreness (Stepping)**					
	**PLA**	**BB**	**statistical analysis**			
Pre	0	0	Time effect, P = <0.001*			
12 h	2.45 ± 2.00	2.14 ± 1.73	*Treatment effect, P = 0.861*			
36 h	3.35 ± 2.25	3.79 ± 1.88	*Interaction, P = 0.903*			
60 h	2.53 ± 1.60	2.65 ± 1.44				

Isometric (ISO), concentric (CON), eccentric (ECC) forces and perceived muscle soreness (stepping) were assessed before (pre) and 12, 36 and 60 hours after 300 eccentric contractions of the quadriceps under control (PLA) or blueberry (BB) smoothie conditions. All values are mean ± standard deviation; * represents nificant (*P* < 0.001) time effect and § a significant *P* < 0.05 treatment (blueberry) x time interaction; n = 10 participants.

### Muscle soreness

Ratings of perceived muscle soreness while stepping up and back down were only taken post-damage (12, 36, and 60 hours) thus comparison from pre-damage values could not be made. While ratings of perceived soreness (RPS) significantly (*p* < 0.0001) differed between subjects (Table 2), no overall difference (*p* = 0.723) was observed between blueberry and control conditions, nor was there any significant (*p* = 0.425) interaction effect between time and treatment. However, subtle recovery differences in RPS between treatments were observed at distinct recovery times after the first values taken 12 hours after the eccentric exercise: the RPS differences between 12 and 36 hours post eccentric exercise were highly significant (*p* = 0.0002) with blueberries, whereas only a slight difference was observed between these two time points in the control condition (*p* = 0.031). Similarly, the RPS values taken after 60 hours recovery were highly significant within the blueberry condition (*p* = 0.008), but once again only slightly differed within the control condition (*p* = 0.049). No correlation was found to exist between muscle soreness and muscle performance recovery (r < 0.09).

### Oxidative stress and inflammatory biomarkers

**Figure 2:**
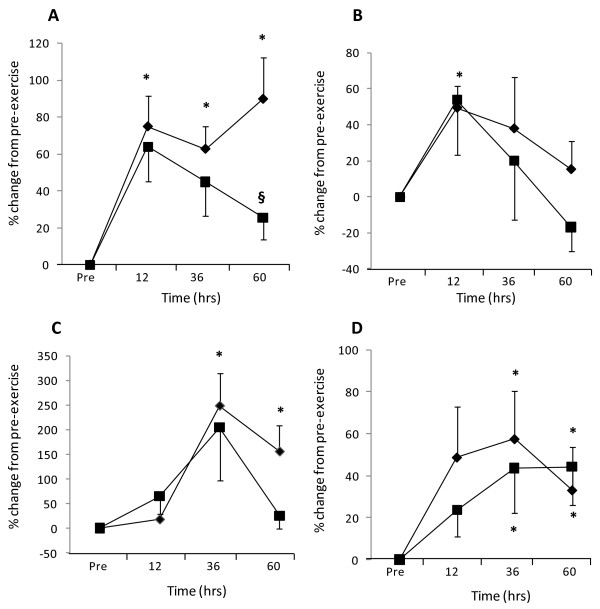
**Modulation of systemic oxidative stress and inflammatory markers after strenuous exercise. [A]** Plasma oxidative capacity, **[B]** protein carbonyls, **[C]** creatine kinase or **[D]** interleukin (IL)-6 were assessed immediately before (pre) and then 12, 36 or 60 hours after 300 eccentric contractions of the quadriceps under control (♦) or blueberry (■) conditions. Results are expressed as mean ± standard error of percentage change from pre-eccentric exercise measurements. * *P* < 0.05 represents significant time difference from pre-exercise levels and § *P* < 0.05 represents significant treatment (blueberry) and time interaction, n = 10 volunteers.

Application of 300 strenuous eccentric contractions to induce fatigue and muscular damage caused an increase in oxidative stress and inflammation in both the blueberry and control beverage conditions. As shown in Figure 2, a significant (*p* < 0.01) increase in plasma oxidative stress markers, ROS-generating potential (Figure 2A) and protein carbonyls (Figure 2B) were observed 12 hours after muscle damage in both conditions. After 36 hours recovery, a gradual decrease in plasma ROS-generating potential (Figure 2A) was observed in the blueberry condition, whereas ROS-generating potential remained elevated in the control condition (*p* < 0.01). A large and significant (*p* < 0.01) increase in plasma carbonyls was observed at 12 hours in both conditions, followed by a gradual decrease (Figure 2B). Although an accelerated decline in plasma carbonyls was observed with blueberries, the difference was not statistically significant (*p* = 0.06). Inflammatory biomarkers associated with muscle damage, CK and IL-6 were measured. A gradual and significant (*p* < 0.05) increase in serum CK (Figure 2C) was observed in both conditions, between pre-exercise and 36 hours after. The CK levels detected following 60 hours recovery were lower in the blueberry beverage condition for the majority (8 out of 10) of the participants, however the overall difference was not significant (*p* = 0.840). In addition, no interaction effect between time and treatment was observed (*p* = 0.426). Assessment of plasma IL-6 (Figure 2D) during the recovery period revealed a gradual increase in plasma IL-6 following exercise. Although this was significantly (*p* < 0.05) different from pre-exercise levels after 36 hours and 60 hours of recovery in both the blueberry and control beverage conditions, no blueberry treatment (*p* = 0.198) or time x treatment interactions (*p* = 0.721) were observed.

### Total antioxidant capacity

**Figure 3:**
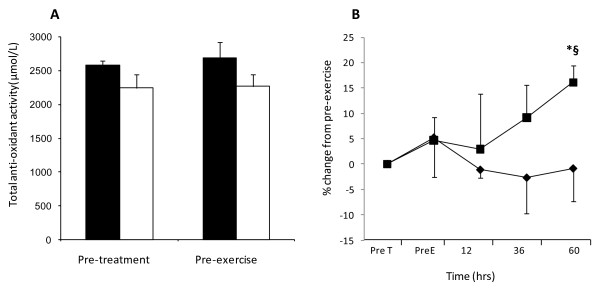
**Plasma total antioxidant potential.** Total antioxidant potential was assessed by the ferric reducing ability of plasma (FRAP) **[A]** before treatment and pre-muscle damaging eccentric exercise in control (filled bars) or blueberry (open bars) groups and **[B]** pre-treatment (preT) at specific times pre (PreE), 12, 36 or 60 hours following 300 eccentric contractions of the quadriceps in control (♦) or blueberry (■) groups. Results are expressed as either mean ± standard error **[A]** FRAP μmol/L or **[B]** % change from pre-treatment values. * *P* < 0.05 represents significant time difference from pre-treatment exercise levels, § *P* < 0.05 represents significant treatment (blueberry) x time interaction, n = 10 volunteers.

The consumption of blueberries had no statistical effect on plasma antioxidant capacity prior to the onset of the eccentric exercise (Figure 3A); control (*p* = 0.140) and blueberry (*p* = 0.149), respectively. However, assessment of plasma antioxidant capacity between the pre-treatment and the 60 hour recovery time point revealed a significant treatment x time interaction (*p* = 0.038). This indicates that consuming the blueberry beverage over the period of the trial increased plasma total antioxidant capacity. Post hoc T-tests revealed no significant difference between the pre-treatment antioxidant values and those measured at the end of the trial in the control group, confirming that plasma antioxidant capacity following strenuous eccentric exercise was only improved by the consumption of the blueberries.

## Discussion

The primary aim of the study was to investigate the effect of blueberry consumption on markers of EIMD and inflammation after strenuous eccentric exercise. By employing a single-leg model, we were able to minimize confounders such as training status, health status, genetics, and lifestyle-relate factors. Further, by closely controlling diet and exercise prior to and during the experimental period, we were able to implement a feeding strategy to successfully explore the effectiveness of New Zealand blueberry consumption on muscle function recovery following strenuous eccentric repetitive quadriceps exercise.

The main findings reveal that consumption of blended New Zealand blueberries at specific times pre and post eccentric muscle damaging exercise accelerates the recovery of muscle peak isometric strength and facilitated a decline in eccentric exercise-induced oxidative stress. The eccentric muscle damaging exercise applied in this study has previously been employed by this group [28,29] and was designed to assess the effectiveness of dietary intervention on the ensuing recovery events. The greatest loss in peak and average torque/tension was seen 12 hours following the 300 maximal eccentric contractions of the quadriceps muscle, indicating muscle damage had been achieved. Indeed, the significant decrease in muscle strength (isometric, concentric and eccentric) observed in both blueberry and control beverage conditions demonstrated that pre-consumption of the blueberry beverage had no treatment effect on the ability of the 300 repetitive eccentric quadriceps muscle contractions to cause the damage and weakness which is expected after a physical effort of this nature. Importantly, in relation to recovery from the 300 eccentric contractions, a significant time-treatment interaction effect on peak isometric tension was observed. Although time-dependent improvement in peak concentric and eccentric muscle strength in the blueberry beverage condition were also evident, they were not statistically significant. While only a small number of subjects were employed in this study, the results support the trend that the consumption fruit, like New Zealand blueberries may expedite recovery in muscle function. For example, similar nutritional interventions trials involving cherry juice [30] or pomegranate-derived ellagitanins [31] have showed an improvement in isometric muscle strength following an eccentric muscle damaging exercise.

The data also indicate that ingestion of a blueberry beverage had no effect on perceived muscle soreness. These observations are similar to other reported in other intervention studies involving fruit [30,31] where an improvement in muscle function, but not pain was reported. In contrast, using a plant phytochemical-protein supplement combination “BounceBack” an improvement in delayed onset muscle soreness was observed independent of exercise-induced inflammation; however, no muscle function performance was reported [32].

Blueberry fruit demonstrate a high antioxidant capacity [14]. The source of this antioxidant capacity is thought to be attributed to the wide range of anthocyanins contained in this fruit and since the vitamin C levels within blueberries are relative low compared to other fruit - the contribution of vitamin C to antioxidant capability is likely to be minor (Table 1). In this study, the effect of vitamin C is also minimized by the addition of a vitamin C fortified apple juice to both the control and blueberry beverages. This resulted in an overall similar antioxidant capacity as determined by ORAC, which further supports the minor contribution of vitamin C. Furthermore our addition of banana to both treatment beverages, which replaced milk (shown to reduce the antioxidant capability of blueberries [21] and dextrose to the control beverage (equivalent to the sugar content found in the blueberry smoothie) ensured that the nutritional and antioxidant capability difference between the control and the blueberry beverage was primarily due to the polyphenolic compounds-derived from the blueberries. Consuming blueberry fruit to enhance plasma antioxidant capacity may be dependent upon what the fruit is consumed with. Serfini *et al.*[21] showed that consumption of 200 g fresh blueberries (the same amount used in this study per serving) in healthy humans caused a transient increase in plasma antioxidant capacity, which was dramatically reduced when the fruit was consumed in conjunction with protein, i.e. a blueberry/milk smoothie. In contrast, Dunlap *et al.*[33] showed no change in plasma antioxidant capacity after two months of feeding blueberries in dogs on a normal healthy diet, whereas Kay and Holub [34] found that humans fed a high fat diet with blueberry fruit had a higher serum antioxidant capacity compared to a control group. In this study, the blueberry beverage was made in the absence of milk and the subjects’ food intake was controlled by provision during the first 24 hours of participation. During the three recovery days, subjects were provided breakfast in the mornings and during the first trial kept a food diary of other food intake (four meals plus snacks). These meals were then replicated during the second trial. Subsequent analysis of food diaries revealed that subjects maintained a similar diet pattern and limited their intake of antioxidant-rich foods as requested. We are therefore confident that the elevation in plasma antioxidant capacity observed following 60 hours of recovery was as a result of the blueberry beverage consumption. It is possible that some sugars in fruit could mediate a control of oxidative stress and the benefits observed in our study. Lotito & Frei reported that phytochemical-rich foods containing some sugars e.g. fructose increased plasma uric acid in human volunteers and contributed to plasma antioxidant status [35]. Dextrose however, was unlikely to be responsible for any effects here as it was utilized in our placebo (equivalent to the sugar content found in the blueberry smoothie) and showed no effects on plasma antioxidant status or control of exercise-induced oxidative stress as reported previously [36].

The 300 repetitive eccentric muscle contractions caused an increase in oxidative stress (ROS-generating potential, protein carbonyls) and inflammatory (CK, IL-6) markers following the eccentric exercise in both experimental conditions. The elevation in these parameters indicates that the strenuous exercise employed in this study is capable of inducing muscle damage (the increase in CK coincided with loss of muscle function in both treatment groups) and that the recovery in muscle function observed by 36 hours in the blueberry condition is independent of the fruit’s inherent antioxidant capacity. Since exercise-induced ROS / inflammation, and especially muscle-derived IL-6 [37] activate down-stream adaptive processes that facilitate skeletal muscle recovery [38], it is feasible that blueberry-derived polyphenolic compounds (such as anthocyanins) may also facilitate these events, which may include the up-regulation of both muscle-specific adaptive processes and overall immunity. It is controversial as to whether an increase in circulating IL-6 correlates with skeletal muscle damage, since eccentric skeletal muscle contraction has been shown to elevate circulating IL-6, as well as other myokines, such as IL-15, IL-8, fibroblast growth factor [37,39], which in turn, have been shown to facilitate anti-inflammatory, energy production and adaptive processes (e.g. anabolic action) and thus facilitate muscle performance and recovery. Although it is quite feasible that the initial increase in circulating IL-6 observed post eccentric exercise in the blueberry condition may be due to skeletal muscle contraction rather than damage, the overall increase in circulating levels of this myokine may serve to promote down-stream muscle recovery events. We have not measured here other myokines or down-stream anti-inflammatory cytokines associated with IL-6 (such as IL-10), but the notion that blueberry consumption facilitates eccentric exercise-induced anti-inflammatory (via IL-6) events is supported by McAulty *et al.*[40] who showed that both acute and long-term blueberry feeding prior to exercise causes an increase in anti-inflammatory cytokines, such as IL-10 and facilitates recovery.

In this study we observed a rapid decline in oxidative stress blood indices that coincided with the increase in plasma antioxidant capacity in the blueberry condition supporting the notion that an increase in plasma antioxidant capacity may be involved in the reduced exercise-induced oxidative stress observed. However, it is currently unclear whether an increase in plasma antioxidant capacity facilitates [41] or hinders the activation of muscle adaptive events aiding muscle recovery. The efficacy of dietary antioxidant supplementation in facilitating recovery following strenuous muscle damaging exercise is under debate. Recent reports indicate that dietary supplements rich in antioxidants, attenuate oxidative stress [42,43], whilst other reports either show that antioxidants have no action [44] or have the ability to induce pro-oxidant effects [45,46]. Moreover, although elevated plasma antioxidant capacity post antioxidant supplementation consumption has been found in many studies [47] have failed to demonstrate an effect or relationship to muscle function recovery following an eccentric exercise-induced damage. Goldfarb *et al.*[11] recently showed that ingestion of whole fruit and/or vegetable extracts may attenuate blood oxidative stress induced by eccentric exercise but no significant effect on functional changes relating to pain and muscle damage were observed. Our findings here concur as all correlations of indices of muscle performance with plasma antioxidant capacity were insignificant; 0.09 and 0.190.

Several studies report the effectiveness of plant-derived phytochemicals at accelerating the recovery from exercise-induced muscle function after damage [30,31]. The health promoting properties of plant-derived phytochemicals are being debated and evidence is building that any benefits are likely independent of their inherent antioxidant capacity [17-20]. Hence it is feasible that polyphenolic compounds derived from blueberries may support muscle repair and recovery through a similar process that is unrelated to the fruit’s antioxidant capacity. Preliminary results from another study we have conducted show that blueberry-derived anthocyanins induce an up-regulation of phase II antioxidant enzymes (unpublished observation) supporting others that report plant-derived anthocyanins activate redox-sensitive transcription factors that lead to the up-regulation of phase II antioxidant enzyme systems [20,48,49]. Since exercise-induced ROS have also been shown to activate redox-sensitive transcription factors - nuclear erythroid-related factor 2 (nrf2) [50,51] and heat shock factor [52], these adaptive mechanisms may serve to facilitate the repair and functional recovery of skeletal muscle. Furthermore, given that anthocyanins also have been described to active the nrf-2 transcription factor [20,48,49] and induce heat shock proteins [52] it is feasible that blueberry-derived anthocyanins may activate similar and/or parallel adaptive mechanisms within damaged muscle and underlie the findings observed here and by others. It is also unclear whether particular anthocyanins or other phytochemicals from fruits (or other sources) are responsible for or synergistic to the benefits reported here. Studies using isolated polyphenolics indicate that they potentially possess diverse functional efficacy within the body, which may not necessarily complement each other. It is feasible that certain fruit species or even certain cultivars (or combinations thereof) may provide the combination of polyphenolics that synergistically act together to most optimally deliver a specific biological action or actions that complement the adaptive events desired by exercise training athletes.

## Conclusions

In conclusion, our study provides evidence that ingestion of a New Zealand blueberry beverage prior to and after eccentric muscle damage accelerates recovery of muscle peak isometric strength, independent of the beverages inherent antioxidant properties. Standardizing blueberry fruit intake based on the lean body mass (g/kg), (assuming that the greater the muscle mass, the greater the force produced during the maximal eccentric protocol [53]) may have given more accurate results. This study has practical implications for all who turn to exercise and dietary antioxidant-rich supplements to maintain their health and performance. It is especially of potential relevance to all athletes who compete over successive days as well as to the general sporting community. Although the literature is divided as to the benefits of antioxidant supplements in affecting the initial muscle damage/inflammation and subsequent recovery of muscle function, this study supports the idea that blueberry consumption induces cellular adaptive events that serve to accelerate muscle repair and recovery of muscle isometric strength. Identifying specific dietary interventions that complement exercise-induced short-term as well as adaptive responses following various exercise strategies (i.e. aerobic exercise-induced oxidative stress or EIMD) may be of greater importance in maintaining health and athletic performance than the consumption of generic dietary supplements based upon their apparent high antioxidant capacity. Follow up studies are therefore warranted with blueberry as a food to assist exercise and should focus upon dose and timing to ascertain important optimum parameters. Of interest also would be studies to determine whether repeated blueberry consumption with a specific eccentric exercise training strategy facilitates recovery between exercise sessions and/or improves the overall performance of athletes in competition.

## Competing interests

All researchers involved in this study have no financial interests concerning the outcome of this investigation.

## Authors’ contributions

YM (with SRS) conceived the idea for the study, contributed to the development of the study design, and primarily responsible for raw data collection. MJB oversaw data collection and statistical analyses, and also led the writing of the manuscript. TM contributed to the development of the study design, raw data collection, and obtainment of ethical approval. SMH, measured and analyzed some of the biochemical parameters and assisted in the development and writing of the draft manuscript. RDH secured funding that assisted with this research and assisted in the development of the study, and in the development and writing of the draft manuscript. SRS (with YM) conceived the idea for the study, obtained funding, led the development of the study design, obtained ethical approval, and assisted in manuscript preparation. All authors read and approved the final manuscript.
